# Supplementary material for: Resolving the full spectrum of human genome variation using Linked-Reads
Source: Genome Res. 2019 Apr;29(4):635–45. doi: 10.1101/gr.234443.118 (PMC6442396; doi:10.1101/gr.234443.118)
Supplement: Supplemental Material [file supp_gr.234443.118_Supplemental_longranger-2.2.zip › longranger-2.2.tar.bz2!/lr2.2-public/lib/stan/cmdstan/stan/doc/api/html/functions_~.html]

Stan: Class Members


|  |  |
| --- | --- |
|  | Stan  2.5.0  probability, sampling & optimization |


- Main Page
- Related Pages
- Namespaces
- Classes
- Files

- Class List
- Class Index
- Class Hierarchy
- Class Members

- All
- Functions
- Variables
- Typedefs
- Enumerator
- Related Functions

- \_
- a
- b
- c
- d
- e
- f
- g
- h
- i
- j
- k
- l
- m
- n
- o
- p
- q
- r
- s
- t
- u
- v
- w
- x
- y
- z
- ~


All Classes Namespaces Files Functions Variables Typedefs Enumerations Enumerator Friends Macros Pages

Here is a list of all class members with links to the classes they belong to:

### - ~ -

- ~accumulator()
  : stan::math::accumulator< T >
- ~adapt\_dense\_e\_nuts()
  : stan::mcmc::adapt\_dense\_e\_nuts< M, BaseRNG >
- ~adapt\_dense\_e\_static\_hmc()
  : stan::mcmc::adapt\_dense\_e\_static\_hmc< M, BaseRNG >
- ~adapt\_diag\_e\_nuts()
  : stan::mcmc::adapt\_diag\_e\_nuts< M, BaseRNG >
- ~adapt\_diag\_e\_static\_hmc()
  : stan::mcmc::adapt\_diag\_e\_static\_hmc< M, BaseRNG >
- ~adapt\_unit\_e\_nuts()
  : stan::mcmc::adapt\_unit\_e\_nuts< M, BaseRNG >
- ~adapt\_unit\_e\_static\_hmc()
  : stan::mcmc::adapt\_unit\_e\_static\_hmc< M, BaseRNG >
- ~argument()
  : stan::gm::argument
- ~base\_hamiltonian()
  : stan::mcmc::base\_hamiltonian< M, P, BaseRNG >
- ~base\_mcmc()
  : stan::mcmc::base\_mcmc
- ~base\_nuts()
  : stan::mcmc::base\_nuts< M, P, H, I, BaseRNG >
- ~base\_static\_hmc()
  : stan::mcmc::base\_static\_hmc< M, P, H, I, BaseRNG >
- ~categorical\_argument()
  : stan::gm::categorical\_argument
- ~chainable()
  : stan::agrad::chainable
- ~chainable\_alloc()
  : stan::agrad::chainable\_alloc
- ~dense\_e\_metric()
  : stan::mcmc::dense\_e\_metric< M, BaseRNG >
- ~diag\_e\_metric()
  : stan::mcmc::diag\_e\_metric< M, BaseRNG >
- ~dump\_reader()
  : stan::io::dump\_reader
- ~dump\_writer()
  : stan::io::dump\_writer
- ~json\_handler()
  : stan::json::json\_handler
- ~list\_argument()
  : stan::gm::list\_argument
- ~prob\_grad()
  : stan::model::prob\_grad
- ~reader()
  : stan::io::reader< T >
- ~sample()
  : stan::mcmc::sample
- ~stack\_alloc()
  : stan::memory::stack\_alloc
- ~stan\_csv\_reader()
  : stan::io::stan\_csv\_reader
- ~unit\_e\_metric()
  : stan::mcmc::unit\_e\_metric< M, BaseRNG >
- ~var\_context()
  : stan::io::var\_context
- ~vari()
  : stan::agrad::vari
- ~writer()
  : stan::io::writer< T >


---

[ Stan Home Page ]

*© 2011–2014,
Stan Development Team.*
